# A Preliminary Study of Uric Metabolomic Alteration for Postpartum Depression Based on Liquid Chromatography Coupled to Quadrupole Time-of-Flight Mass Spectrometry

**DOI:** 10.1155/2019/4264803

**Published:** 2019-04-24

**Authors:** Li Zhang, Wei Zou, Yi Huang, Xiaoke Wen, Jianxi Huang, Yichao Wang, Xiaoqi Sheng

**Affiliations:** NHC Key Laboratory of Birth Defects Research, Prevention and Treatment, Hunan Provincial Maternal and Child Health Care Hospital, Changsha, China

## Abstract

Postpartum depression affects about 10-20% of newly delivered women, which is harmful for both mothers and infants. However, the current diagnosis of postpartum depression depends on the subjective judgment of a practitioner, which may lead to misdiagnosis. Hence, an appended objective diagnosis index may help the practitioner to improve diagnosis. A metabolomic study can find biomarkers as an objective index to facilitate disease diagnosis. Forty-nine postpartum depressed patients and 50 healthy controls were recruited into this study. The metabolites in urine were scanned with LC-Q-TOF-MS. The metabolomic data were analyzed with a multivariate statistical analysis method. Data from 40 patients and 40 controls were used for partial least square-discriminate analysis (PLS-DA). The urine metabolomic profiles of patients were different from those of controls. The PLS-DA model was validated by a permutation test, and the model could accurately classify the other 9 patients and 10 controls in T-prediction. Ten differentiating metabolites were found as main contributors to this difference, which are involved in amino acid metabolism, neurotransmitter metabolism, bacteria population, etc. Some of these potential biomarkers, such as 4-hydroxyhippuric acid, homocysteine, and tyrosine, showed relatively high sensitivities and specificities. The metabolic profile alteration induced by postpartum depression was found, and some of the differentiating metabolites may serve as biomarkers to facilitate the diagnosis of postpartum depression.

## 1. Introduction

Postpartum depression occurs in the first few weeks after delivery, as specified in the International Classification of Diseases, 10th edition (ICD-10). As a common disease, postpartum depression affects about 10-20% of newly delivered women [1, 2], which is deemed as a great threat as it is associated with low mood, sadness, decreased maternal sensitivity and attachment to infant, poor child development, and increased risk of suicide and infanticide [3–5]. The risk of suicide, owing to postpartum depression, is relatively high between all depressed women, and about 5% to 14% of postpartum depressed patients have self-harm ideation, which is more than prevalent attempts or actual death [3]. On the other hand, the mother is the first social environment for infant and introduces infants to the outside world, and hence, a bad mother-child relationship produced by postpartum depression will interfere with the healthy development of the emotion, behavior, and cognition of the infant [6]. A comprehensive study showed that children brought by postpartum depression mothers performed worse cognitive abilities at 18 months of age, comparing to the children with mentally healthy mothers [7].

The etiology of postpartum depression is complex. Clinical (premature births, intrauterine growth impairment, operative deliveries, and admission to neonatal care units), biological (sleep disruption, deregulation of neurotransmitters, and serotonin), hormonal (thyroid, cortisol, and oxytocin), and psychological variables (stressful life events, marital conflict, and low social support, attachment insecurity, and personality features) contribute to the development of postpartum depression [8–11]. A clinician subjectively diagnoses this disease according to the symptomatic clusters and scales, which is the sole diagnostic method. In this case, the accuracy of this diagnosis highly relies on the academic knowledge and clinical experience of the clinician. Currently, a certain part of depressed patients is not accurately diagnosed [12, 13]. Besides, the pronounced common accompanied physical symptoms, such as debilitation and dizziness, may lead to the negligence of the depression symptom for patients [14]. Therefore, biomarkers, obtained from a laboratory-based test, may be helpful for the diagnosis of postpartum depression.

Through quantification of the metabolites in biofluids, such as the urine and blood, metabolomic studies show the characteristic of metabolism *in vivo* and illuminate the physiology condition, which can find differentiating metabolites (biomarkers) for disease diagnosis. Nowadays, a large number of clinical metabolomic studies have been carried out to explore metabolic biomarkers [15]. Especially, metabolomic studies on the cerebrospinal fluid, blood, and urine of major depressive disorder patients have showed the metabolic alterations and found potential biomarkers [16–19]. As postpartum depression occurs in the immediate postpartum period, the patients are considered to be biochemically different from the other depressions patients [20] and hence may show different metabolomic alterations. Therefore, the metabolomic study on postpartum depression is necessary. However, reports on metabolomics of postpartum depression are rare. Recently, a gas chromatography-mass spectroscopy (GC-MS)-based uric metabolomic study on postpartum depression has been carried out and found several differentiating metabolites [21]. As the liquid chromatography coupled to quadrupole time-of-flight mass spectrometry (LC-Q-TOF-MS), compared with GC-MS, can detect different metabolites, an LC-Q-TOF-MS-based uric metabolomic study was carried out to find out new metabolomic alterations associated with postpartum depression. The potential biomarkers found in this study may facilitate the diagnosis of this disease in the future.

## 2. Materials and Methods

### 2.1. Chemicals and Reagents

Acetonitrile and methanol (LC grade) were from Fisher Scientific (Fair Lawn, NJ, USA). Milli-Q (Bedford, MA, USA) pure water was used in this experiment. Formic acid and ammonium acetate (LC-grade), stearoylethanolamide, homocysteine, methylmalonic acid, 4-hydroxyhippuric acid, tyrosine, vanillylmandelic acid, glutaric acid, alanine, and naringin were obtained from Sigma-Aldrich (St. Louis, MO, USA).

### 2.2. Subjects

This study was approved by the Ethical Committee of Hunan Provincial Maternal and Child Health Care Hospital (No. EC201507), according to the Declaration of Helsinki. Informed consent was obtained from all individual participants included in the study. All participants, aged 20-40 years, were enrolled from the Hunan Provincial Maternal and Child Health Care Hospital from April to December in 2016. Postpartum depressed patients were enrolled from the Department of Clinical Psychology, who were at the sixth week after delivery. The patients were diagnosed with postpartum depression with a single depressed episode based on a psychiatric interview by two psychiatrists according to the affective module of ICD-10. Depressive symptoms were assessed by the 17-item Hamilton Depression Rating Scale (HDRS) [22], and only depressed subjects with HDRS scores greater than 17 were recruited. Healthy control subjects were recruited from the postnatal examination center when they received routine health examination at the sixth week after delivery. The 17-item HDRS was used to screen the women's depressive symptoms. Women who scored below cut-off point 7 on HDRS were recruited into the healthy control group. The others whose scores were not less than 7 were transferred to the Department of Clinical Psychology for further assessment. All candidates with any previous lifetime history of neurological and/or ICD-10 classification of mental and behavioral disorders were excluded from this study. All patients and controls who suffered systemic illness or had received any kind of pharmacologic treatment in the last month were also excluded. All participants were randomly divided into the training group for multivariate statistical analysis and the validation group for T-prediction.

The urine sample was collected in the morning after fasting overnight and stored at −80°C.

### 2.3. Urine Sample Preparation

Urine samples were thawed at 4°C. A mixture of 50 *μ*l urine and 50 *μ*l water was added with 300 *μ*l MeOH/acetonitrile solution (1 : 1, *v*/*v*). The internal standard naringin was added to the sample, and the sample was stored at 4°C overnight. Then, the sample was centrifuging for 10 min at 13,000 rpm. 5 *μ*l supernatant was injected into the LC-Q-TOF-MS system. Quality control (QC) samples were prepared using mixed urine.

### 2.4. LC-Q-TOF-MS Analysis

The LC-Q-TOF-MS system used in this study was consisted by a 1290 LC and a 6540 mass system (Agilent Technology, USA). The chromatographic column was ZORBAX Eclipse XDB-C18 (3.0 × 150 mm i.d., 3.5 *μ*m) (Agilent Technology, USA). Acetonitrile (A) and 0.1% formic acid with 5 mmol/l ammonium acetate (B) were used as the mobile phase. The gradient was 5% A at *t* = 0 min, changed linearly to 100% A from *t* = 2 min to *t* = 6 min and hold for 4 min, with a flow rate of 0.4 ml/min. The column temperature was set at 35°C. The mass parameters in positive and negative ion modes were as follows: capillary voltage 3500 V, nozzle voltage (Expt) 500 V, fragmentor 120 V, skimmer 65 V, gas temperature 250°C, sheath gas temperature 350°C, collision energy 20 V (MS/MS mode), and scan range *m*/*z* 20–1250. Both drying gas and collision gas were nitrogen.

### 2.5. Data Analysis

Both internal standards and QC sample were used to control the quality of sample analysis. Profinder B08.00 software (Agilent Technology, USA) was used for data extraction and alignment. Peak areas of metabolites were normalized by that of creatinine. Subsequently, to remove the average concentration difference between different metabolites which may lead to the bias in finding differentiating metabolites, the normalized peak area of each metabolite underwent mean centering. Then, a dataset including detected substances with corresponding normalized/mean-centered peak area was produced for further statistical analysis. To discriminate the metabolomic profiles between patients and controls, partial least square-discriminate analysis (PLS-DA) was performed by SIMCA-P 11.5 (Umetrics, Sweden) whose instruction can be obtained from https://umetrics.com/. With the SIMCA-P software, permutation analysis (200 times) and T-prediction were performed to validate the PLS-DA model. During the procedure of T-prediction, unknown samples in the prediction group were statistically analyzed under the built PLS-DA model for prediction or discrimination. The variable importance in the projection (VIP) values of metabolites was generated from the PLS-DA model. The *P* values were obtained from a *t*-test. To control the false discovery rate, the *Q* values were calculated by correcting *P* values with Benjamini and Hochberg's procedure [23]. Metabolites with VIP > 1 and *Q* < 0.05 were deemed as potential biomarkers. Then, these metabolites were identified by their accurate molecular weights and fragment patterns referring to the Metlin database from Agilent, and the further identification resorted to the standards if commercially available. The volcano plot from Metaboanalysis 4.0 (http://www.metaboanalyst.ca) was to show the fold change (FC) and *P* values of substances, and the heatmap was to visualize the level changes of potential biomarkers. The receiver operator characteristic (ROC) curve of each metabolite was plotted with the sensitivity on the *y*-axis and the corresponding false-positive rates on the *x*-axis, with a 95% confidence interval (CI). Unpaired Student's *t*-test was performed on SPSS 16.0 (SPSS Inc., Chicago, USA) for statistical analysis. *P* < 0.05 was considered statistically significant.

## 3. Results

### 3.1. Information of Subjects

There are 49 postpartum depressed patients and 50 mentally healthy controls participated in this study. The metabolomic data of 40 patients and 40 controls were used for multivariate statistical analysis, and the data of the remaining 9 patients and 10 controls were employed for T-prediction. As presented in Table 1, demographics, age, race, and body mass index (BMI) of patients and controls showed no significant difference. The HDRS scores of patients were significantly different from that of controls.

### 3.2. LC-Q-TOF-MS Analysis of Urine

Six QC samples were prepared and analyzed to test the repeatability of this method. The retention times and peak areas of selected 3 substances in each ion mode were statistically analyzed. Retention time shifts of these substances were less than 0.2 min, the relative standard deviations (RSDs) were less than 1.6%, and the RSDs of peak areas were less than 8.5% (data not presented). These results showed a good repeatability of this method. The internal standard naringin in samples was employed to monitor the sample preparation and instruction condition. The RSD of its peak areas in all urine samples was 9.2%, indicating a good availability of this metabolomic analysis. After data extraction by Profinder B08.00 software, 485 substances detected in each urine sample were used for multivariate statistical analysis.

### 3.3. Multivariate Statistical Analysis

**Figure 1:**
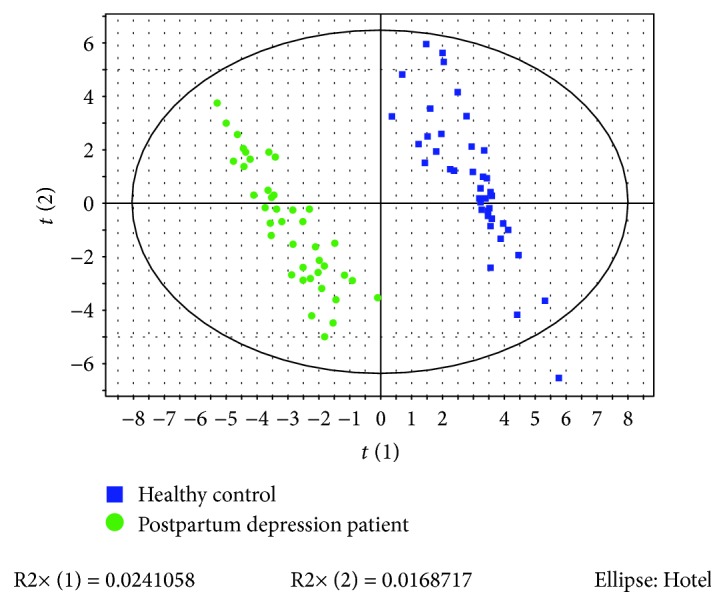
PLS-DA model (R2X = 0.041, R2Y = 0.968, and Q2 = 0.398) discriminating the metabolic profiles of postpartum depressed patients and healthy controls.

The PLS-DA was carried out to discriminate the metabolomic profiles of patients and controls. As showed in Figure 1, the metabolomic profiles of patients and controls were distributed into two separated areas.

**Figure 2:**
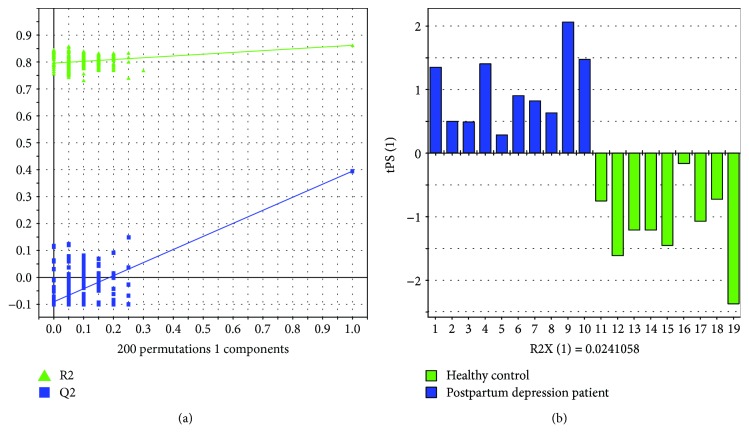
Validation of the PLS-DA model by the 200-time permutation test (a) and T-prediction (b).

A 200-time permutation test was performed to validate the PLS-DA model. As showed in Figure 2(a), all the left Q2 values by permutations were lower than the right original Q2 value, which meant that the model was valid. A T-prediction was carried out to further validate the PLS-DA model, within the further 9 patients and 10 controls. The results showed that the PLS-DA model could accurately predict these 19 samples (Figure 2(b)).

**Figure 3:**
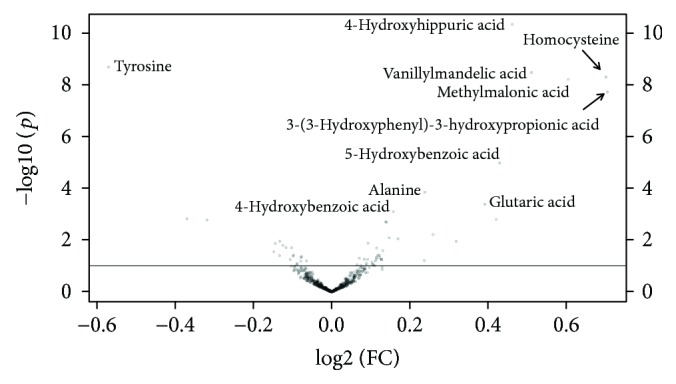
Volcano plot presenting the -log10(*P*) and log2(FC) of metabolites.

The volcano plot of detected substances was presented in Figure 3, and 10 potential biomarkers whose VIP > 1 and *Q* < 0.05 were marked in this plot. All these metabolites were identified by referring to the Metlin database, and 4-hydroxyhippuric acid, 4-hydroxybenzoic acid, alanine, glutaric acid, homocysteine, methylmalonic acid, tyrosine, and vanillylmandelic acid were further identified by commercial standards. The chromatography and mass spectra information, VIP, FC, *P*, and *Q* values of these potential biomarkers are summarized in Table 2.

**Figure 4:**
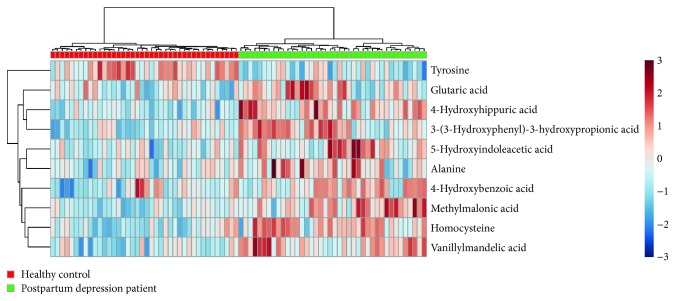
Heatmap of the unsupervised hierarchical clustering of 10 differentiating metabolites across the postpartum depressed patients and healthy controls. The columns indicate the subjects, and the rows indicate the differentiating metabolites.

To visualize the level changes of these potential biomarkers in postpartum depression patients, a heatmap is plotted and presented in Figure 4. The concentration of tyrosine in urine of patients was significantly lower than that of controls, and the level of the remaining differentiating metabolites showed a reverse tendency.

### 3.4. Potential Biomarker Evaluation

The ROC curves of potential biomarkers, obtained from biomarker analysis in MetaboAnalyst 3.0, were plotted to evaluate their potentials in serving as biomarkers for postpartum depression diagnosis. The AUCs of ROC curves in 95% CI, sensitivities, and specificities of these 10 potential biomarkers are summarized in Tables 2 and 3. Homocysteine, 4-hydroxyhippuric acid, and tyrosine showed relatively high sensitivities and specificities and were combined with logistic regression in SPSS software. The logistic regression model is logit (*P*) = –24.423 − 0.11 × tyrosine + 0.247 × 4-hydroxyhippuric acid + 0.102 × homocysteine, where logit (*P*) is the probability of postpartum depression. The AUC (95% CI), sensitivity, and specificity for the combined metabolites are 0.995 (0.981-1.000), 0.938, and 0.980, respectively.

## 4. Discussion

Postpartum depression is a serious psychiatric disorder, as it could be detrimental for both the mother and the child. Currently, the diagnosis of postpartum depression primarily depends on the subjective judgment of the clinician, according to the symptom of the patient. Besides, accompanied physical symptoms may lead to the negligence of the depression symptom for patients. Biomarker, as an objective index based on a laboratory test, may help the current diagnosis of postpartum depression. In this metabolomic study with an LC-Q-TOF-MS method, 10 differential metabolites in urine were found as potential biomarkers for facilitating the diagnosis. These metabolites indicated that postpartum depression could lead to alterations of amino acid metabolism, neurotransmitter metabolism, bacteria population, etc. Among them, alterations of neurotransmitter metabolism and bacterial metabolism in this study have not been found in the former GC-MS-based study [21].

In this study, the concentration of homocysteine in urine was significantly elevated in postpartum depressed patients, which was in concurrence with the previous findings in depressed patients [24, 25]. The restriction of homocysteine metabolism may lead to inhibition of methylation reaction in vivo which will affect the composition of phospholipids, the structure of cell membranes, and receptor function [26]. Inhibited methylation of myelin basic protein can lead to loosely compacted myelin and decreased function of nerve conduction [27]. Besides, homocysteine has been proved to interact with transition metals to show the abilities of oxidation toxicity and neuronal cell damage [28]. Vitamin B_12_ is essential in the central nervous system, as it takes part in the methylation response which is essential for the production of monoamine, neurotransmitters, phospholipids, and nucleotides. Deficiency of vitamin B_12_ may lead to an impaired methylation and the following psychiatric disease [29], such as depression [30]. Methylmalonic acid is a metabolic product of vitamin B_12_, whose high level indicates a deficiency of vitamin B_12_ [31]. In this study, we found the elevated level of methylmalonic acid in postpartum depressed patient, supporting the previous researches. Here, the levels of 4-hydroxyhippuric acid and 3-(3-hydroxyphenyl)-3-hydroxypropionic acid in postpartum depressed patients were higher than that in healthy women, which were observed in autism children [32, 33]. 3-(3-Hydroxyphenyl)-3-hydroxypropionic acid is a metabolic product from the action of the bacterium *in vivo*, whose level elevation may suggest the disturbance of microbial population balance. Tyrosine is a precursor of the catecholamine of the brain. The level of tyrosine was decreased in the urine of postpartum depressed patients in this study, which was consistent with the previous studies that showed the decrease in the blood of depressed patients [34] and supported the hypothesis that dopaminergic factors are involved in clinical depression [35]. 5-Hydroxyindoleacetic acid is a breakdown product of serotonin, whose increase in this study might indicate an extensive consumption of serotonin. The increase of 5-hydroxyindoleacetic acid was also observed in the cerebrospinal fluid of major depressed patients [36]. Vanillylmandelic acid is a major metabolic product of norepinephrine which is involved in the noradrenergic system and the hypothalamic-pituitary-adrenal axis, whose elevation is related to the depression symptom [37, 38] and was also found in this study. Glutaric acid is mainly produced from the metabolism of lysine, whose fluctuation in postpartum depressed patients indicates the change of lysine metabolism. Alanine, as a nonessential amino acid, is an inhibitory neurotransmitter in the brain. The concentration of alanine in postpartum depressed patients was significantly higher than that in controls in this study, which was consistent with that found in other depressed patients [39].

A meta-analysis pooling 50,371 patients across 41 studies to assess the diagnosis of depression showed that only 47.3% of cases were correctly identified by practitioners; across 19 studies, the sensitivity was 50.1% and the specificity was 81.3% at one-off assessment [12]. In this study, some of potential urine biomarkers, such as 4-hydroxyhippuric acid, homocysteine, and tyrosine showed relatively high sensitivities and specificities. The sensitivity and specificity for the combined metabolites are 0.938 and 0.980, respectively, which might be satisfactory for clinic use. Hence, these potential biomarkers might offer a novel objective diagnosis method of postpartum depression and help practitioners to improve diagnosis. Nevertheless, there are some limitations within this study. Firstly, only about 50 participants were recruited into the patient group and control group, respectively, and hence, this study is just a preliminary study. Secondly, the mothers mainly come from Hunan Province, China, and whether these potential biomarkers could be used in other human races is unknown. Moreover, these differentiating metabolites could discriminate the postpartum depressed patients from healthy mothers, but whether they could discriminate postpartum depression from other neuropsychiatric disorders, such as postpartum psychosis, postpartum bipolar, and baby blue, is unclear. Therefore, further cohort studies should be carried out to clarify these problems.

## 5. Conclusion

A postpartum depression urine metabolomic study was carried out by using the LC-Q-TOF-MS method. The urine metabolomic profile of postpartum depressed patients could be discriminated from that of healthy mothers. Ten differentiating metabolites were found as potential biomarkers to facilitate the diagnosis of postpartum depression, which are involved in amino acid metabolism, neurotransmitter metabolism, bacteria population, etc.

## Figures and Tables

**Table 1:** Demographic characteristics of the study population.

Parameters	Primary cohort			Validation cohort		
	PDP^a^	HC^b^	*P* value	PDP	HC	*P* value
Number of cases	40	40	—	9	10	—
Mean age (y), mean (SD)	27.0 (4.6)	27.2 (4.7)	0.831	24.7 (4.0)	27.7 (4.9)	0.161
Racial origin: Asian (Chinese) (*n* (%))	40 (100)	40 (100)	—	9 (100)	10 (100)	—
Body mass index	26.2 (3.8)	25.2 (3.3)	0.192	25.8 (3.2)	24.9 (2.1)	0.433
HDRS scores	29.5 (8.8)	4.3 (1.6)	<0.001	27.9 (7.1)	4.3 (1.4)	<0.001

^a^Postpartum depressed patients. ^b^Healthy controls.

**Table 2:** The differentiating metabolites contributing to the discrimination of urine metabolomic profiles of postpartum depressed patients and healthy controls in the training group.

Metabolite	RT (min)	Ion mode	MS (*m*/*z*)	MS/MS (*m*/*z*)	VIP value	*P* value	*Q* value	Trend	Fold change^c^	AUC (95% CI)	Sensitivity	Specificity
Alanine	1.7	[M+H]^+^	90.0557	44	2.97	<0.001	0.007	Up^a^	1.18	0.722 (0.621-0.827)	0.675	0.675
Methylmalonic acid	1.4	[M-H]^−^	117.0191	73	4.28	<0.001	<0.001	Up	1.52	0.848 (0.744-0.918)	0.725	0.825
Homocysteine	3.4	[M+H]^+^	136.0427	118, 90	4.30	<0.001	0.012	Up	1.62	0.852 (0.770-0.931)	0.775	0.800
Tyrosine	3.3	[M+H]^+^	182.0804	165, 136, 91	4.38	<0.001	0.048	Down^b^	-1.48	0.859 (0.761-0.920)	0.700	0.850
Glutaric acid	1.6	[M-H]^−^	131.0346	87, 59	2.77	<0.001	0.019	Up	1.31	0.711 (0.587-0.816)	0.625	0.775
Vanillylmandelic acid	1.9	[M-H]^−^	197.0450	137	4.33	<0.001	0.029	Up	1.42	0.851 (0.762-0.918)	0.750	0.750
4-Hydroxyhippuric acid	3.8	[M-H]^−^	194.0461	150, 93	4.71	<0.001	<0.001	Up	1.38	0.884 (0.806-0.946)	0.825	0.825
4-Hydroxybenzoic acid	3.6	[M-H]^−^	137.0231	93	2.64	<0.001	0.033	Up	1.11	0.714 (0.599-0.831)	0.650	0.725
5-Hydroxyindoleacetic acid	4.9	[M+H]^+^	192.0647	146, 118	3.39	<0.001	<0.001	Up	1.35	0.752 (0.650-0.836)	0.725	0.650
3-(3-Hydroxyphenyl)-3-hydroxypropionic acid	2.2	[M-H]^−^	181.0499	137, 119	4.16	<0.001	<0.001	Up	1.63	0.827 (0.725-0.907)	0.825	0.725

RT: retention time; VIP: variable importance in the projection; CI: confidence interval. ^a^The level in patients is higher than that in controls. ^b^The level in patients is lower than that in controls. ^c^The fold change was calculated by dividing the level of metabolite in patients by that in controls.

**Table 3:** The ROC curve of the differentiating metabolites in the validation group.

Metabolite	AUC (95% CI)	Sensitivity	Specificity
Alanine	0.733 (0.478-0.944)	0.778	0.700
Methylmalonic acid	0.833 (0.600-0.989)	0.667	1.000
Homocysteine	0.856 (0.644-1.000)	0.778	0.900
Tyrosine	0.867 (0.689-0.989)	1.000	0.700
Glutaric acid	0.667 (0.367-0.889)	0.667	0.600
Vanillylmandelic acid	0.756 (0.467-0.933)	0.556	0.900
4-Hydroxyhippuric acid	0.889 (0.678-1.000)	0.778	0.900
4-Hydroxybenzoic acid	0.656 (0.311-0.900)	0.556	0.900
5-Hydroxyindoleacetic acid	0.700 (0.400-0.922)	0.667	0.800
3-(3-Hydroxyphenyl)-3-hydroxypropionic acid	0.722 (0.500-0.911)	0.667	0.800

## Data Availability

The data used to support the findings of this study are available from the corresponding author upon request.
